# Genetic diversity, population structure, and effective population size in two yellow bat species in south Texas

**DOI:** 10.7717/peerj.10348

**Published:** 2020-11-18

**Authors:** Austin S. Chipps, Amanda M. Hale, Sara P. Weaver, Dean A. Williams

**Affiliations:** 1 Department of Biology, Texas Christian University, Fort Worth, TX, United States of America; 2 Biology Department, Texas State University, San Marcos, TX, United States of America; 3 Bowman Consulting Group, San Marcos, TX, United States of America

**Keywords:** Bats, *Dasypterus ega*, *Dasypterus intermedius*, * Lasiurus*, Microsatellites, Mitochondrial DNA, Population genetics, Tree bats, Wind energy development, Wind power

## Abstract

There are increasing concerns regarding bat mortality at wind energy facilities, especially as installed capacity continues to grow. In North America, wind energy development has recently expanded into the Lower Rio Grande Valley in south Texas where bat species had not previously been exposed to wind turbines. Our study sought to characterize genetic diversity, population structure, and effective population size in *Dasypterus ega* and *D. intermedius*, two tree-roosting yellow bats native to this region and for which little is known about their population biology and seasonal movements. There was no evidence of population substructure in either species. Genetic diversity at mitochondrial and microsatellite loci was lower in these yellow bat taxa than in previously studied migratory tree bat species in North America, which may be due to the non-migratory nature of these species at our study site, the fact that our study site is located at a geographic range end for both taxa, and possibly weak ascertainment bias at microsatellite loci. Historical effective population size (N_EF_) was large for both species, while current estimates of Ne had upper 95% confidence limits that encompassed infinity. We found evidence of strong mitochondrial differentiation between the two putative subspecies of *D. intermedius* (*D. i. floridanus* and *D. i. intermedius*) which are sympatric in this region of Texas, yet little differentiation using microsatellite loci. We suggest this pattern is due to secondary contact and hybridization and possibly incomplete lineage sorting at microsatellite loci. We also found evidence of some hybridization between *D. ega* and *D. intermedius* in this region of Texas. We recommend that our data serve as a starting point for the long-term genetic monitoring of these species in order to better understand the impacts of wind-related mortality on these populations over time.

## Introduction

Bats face significant threats from a variety of sources including habitat loss, diseases like white-nose syndrome, human disturbance and persecution, and climate change (Jones et al., 2009; O’Shea et al., 2016; Frick, Kingston & Flanders, 2019). The growth of wind energy development, which is critical for reducing greenhouse gas emissions and mitigating the effects of climate change, unfortunately also represents a potential threat to the persistence of bat populations as bats are killed at wind energy facilities worldwide (Arnett & Baerwald, 2013; Arnett et al., 2016; Zimmerling & Francis, 2016; Thaxter et al., 2017). In the United States and Canada, most of the research investigating bat mortality at wind energy facilities has focused on three bat species: *Aeorestes* [*Lasiurus*] *cinereus*, *Lasiurus borealis*, and *Lasionycteris noctivigans*, as they comprise the majority of fatalities reported annually (Arnett & Baerwald, 2013; Smallwood, 2013; Zimmerling & Francis, 2016). All three species are migratory, solitary, and tree-roosting; and as such, are dispersed across the landscape making it impossible to estimate census population sizes or monitor population trends using traditional mark-recapture methods (Schorr, Ellison & Lukacs, 2014). Given this challenge and the lack of empirical demographic data, studies of these three species have implemented population genetics methods to better understand genetic diversity and population connectivity (Luikart et al., 2010), which in turn likely affect species’ resiliency to sustained wind turbine mortality. From the studies published to date, these migratory tree bats all have high genetic diversity, large effective population sizes, and show no evidence of population sub-structure (Korstian, Hale & Williams, 2015; Vonhof & Russell, 2015; Pylant et al., 2016; Sovic, Carstens & Gibbs, 2016), most likely due to individuals mating during annual migration resulting in high gene flow among populations.

Given that population genetics methods can be implemented in repeated sampling efforts to detect evidence of population declines over time (Schwartz, Luikart & Waples, 2007; Antao, Pérez-Figueroa & Luikart, 2011), genetic data collected from bat species killed at wind turbines have the potential to reduce uncertainty regarding the impacts of wind energy on bat populations, and could be an important component of long-term monitoring efforts for conservation. Furthermore, the opportunity for expanding this approach beyond the three migratory tree bats mentioned above is high given the annual abundance of bat carcasses that could be available for DNA collection (Arnett & Baerwald, 2013; AWWI, 2018). The large quantity of bat carcasses salvaged from wind energy facilities at distinct geographic locations at known times of the year provides an opportunity to answer questions related to population status, cryptic species, geographic ranges, and seasonal movements—all aspects of bat biology that warrant additional investigation. With the population status of many bat species worldwide being unknown (Frick, Kingston & Flanders, 2019), fatalities at wind energy facilities in North America provide an opportunity to indirectly estimate the population-level effects of wind-related mortality if repeated genetic sampling efforts are carried out over time (Schwartz, Luikart & Waples, 2007).

Recent wind energy expansion into the Lower Rio Grande Valley of Texas has led to two additional bat species, the northern yellow bat (*Dasypterus intermedius*) and the southern yellow bat (*D. ega*), being identified as collision fatalities at wind turbines (AWWI, 2018). Yellow bats are Lasiurine bats and are therefore closely related to hoary bats (*Aeorestes*) and red bats (*Lasiurus*; Baird et al., 2015; Baird et al., 2017). Thus, the potential for collision mortality from wind energy development is high for these species given their shared life histories and the level of mortality seen annually in *A. cinereus* and *L. borealis*. The purpose of our study was to use *Dasypterus* carcasses salvaged from two wind energy facilities located in far-south Texas to provide insights into aspects of yellow bat basic biology such as seasonal movements, population connectivity, and life-history characteristics. Specifically, we sought to provide contemporary estimates of genetic diversity, effective population size, and population structure which can be used as a baseline for the long-term genetic monitoring of these species, and provide recommendations for future research. And finally, due to the location of our study site, we assessed the status of *D. intermedius* subspecies designations (*D. intermedius floridanus* and *D. i. intermedius*; Webster, Jones & Baker, 1980) to determine whether the groups are genetically distinct and if both putative subspecies are sympatric in south Texas.

## Materials & Methods

### Focal species

Overall, the geographic range of *D. ega* is expansive, encompassing much of South America, Central America, and the southern region and gulf coast of Mexico (Barquez & Diaz, 2016). In contrast, the range of *D. intermedius* is more limited in scope and includes the southeastern U.S., continuing west along the Gulf of Mexico as far south as Nicaragua, and then extends northward along the Pacific coast of Mexico (Miller & Rodriguez, 2016). Within Texas, both *D. ega* and *D. intermedius* have a limited geographic range (Ammerman, Hice & Schmidly, 2012; see Fig. 2 in Decker et al., 2020). The northern limit to the range of *D. ega* was previously thought to include only the southernmost counties of Texas, although a recent study documents a northern expansion into south-central Texas (Decker et al., 2020). *D. intermedius* was known primarily from Texas counties along the Gulf of Mexico, but now appears to be expanding inland (Decker et al., 2020). Previous research separated *D. intermedius* into two subspecies based on body size and pelage color, and suggested that they differentiated during the Last Glacial Maximum (LGM) in separate refugia (Hall & Jones Jr, 1961). Today *D. i. intermedius* is believed to occur from Central America into southern Texas, whereas *D. i. floridanus* occurs from Florida along the Gulf Coast and into southern Texas. Several recent studies have revealed that these two subspecies are clearly differentiated at mitochondrial loci and that they are sympatric in southern Texas (Chipps et al., 2020; Decker & Ammerman, 2020). Decker & Ammerman (2020) estimated that these subspecies differentiated long before the LGM (∼5.5 Ma) and found strong mito-nuclear discordance using a nuclear intron (chymase intron 1). Decker & Ammerman (2020) further suggest that secondary contact and interbreeding between these taxa is the primary cause of the discordance; a result that is also supported by evidence of intergradation in morphology across their range (Hall & Jones Jr, 1961).

### Sample collection

We obtained wing tissue samples from *D. ega* and *D. intermedius* carcasses collected during post-construction fatality surveys at wind energy facilities in Starr and Hidalgo Counties (Texas) from March through November of 2017 and 2018 (*n* = 439 carcasses; Weaver, 2019; Weaver et al., 2020). Duke Energy and EDP Renewables provided access to their wind energy facilities (EDPR: contract number 0320007188). Bat carcasses were collected in accordance with the Texas State University Institutional Animal Care and Use Committee (IACUC: permit number 20171185494) and Texas Parks and Wildlife Department (TPWD: permit number SPR-0213-023). Wing tissue samples were stored in vials containing 95% ethanol. We extracted DNA from the preserved tissue samples following the ammonium acetate/isopropanol precipitation method detailed in Korstian et al. (2013). We used DNA barcoding to confirm or correct species identification (Chipps et al., 2020).

### Mitochondrial DNA sequencing

We sequenced DNA extracted from all wing tissue samples at a 550 bp section of the mitochondrial cytochrome *c* oxidase I (COI) gene. To amplify the COI gene using a polymerase chain reaction (PCR), we used an M13-tailed primer cocktail (Ivanova et al., 2007); cocktail 2 in Clare et al., 2007). PCR reactions (10 µL) contained 10–50 ng DNA, 0.2 µM of the primer cocktail, 1X BSA, and 1X AccuStart™ II PCR SuperMix. PCR reactions were completed using an ABI 2720 thermal cycler with parameters: one cycle at 94 °C for 15 min, followed by 30 cycles of 30 s at 94 °C, 90 s at 57 °C, 90 s at 72 °C, and then a final extension of 5 min at 72 °C. Products were sequenced using ABI Big Dye Terminator Cycle Sequencing v3.1 Chemistry (Applied Biosystems, USA) with the PCR primers. DNA sequences were analyzed on an ABI 3130XL Genetic Analyzer (Applied Biosystems, USA); trimmed, edited and assembled into contigs using Sequencher v5.1 (Gene Codes, USA); and then aligned in MEGA v10 (Kumar et al., 2018). Aligned sequences were translated to verify the absence of stop codons, after which they were compared to GenBank voucher sequences to generate a species ID. Only sequences > 400 bp in length were used and our criterion to accept a molecular species identification required an identity value > 98% in BLAST. Unique sequence haplotypes were detected using GenAlEx v6.5 (Peakall & Smouse, 2006).

### Nuclear microsatellite loci amplification

We amplified 118 *D. ega* and 262 *D. intermedius* samples at 13 microsatellite loci in three groups: multiplex A with primers: Coto_G12, LAS7468, LAS8830, LAS9555 and LAS9618; multiplex B with primers: Cora_F11, LAS2547, LAS8425, LAS9151 and LbT; and multiplex C with primers: LAS7831, LcM, LcU. Primers were previously developed for use in *L. borealis*, *A. cinereus*, and *Corynorhinus* spp. by Piaggio, Figueroa & Perkins (2009), Piaggio et al. (2009), Korstian, Hale & Williams (2014) and Keller et al. (2014). PCR reactions were performed using the same ratios of reagents as mitochondrial sequencing, but had cycling parameters of: one cycle at 94 °C for 15 min, followed by 30 cycles of 30 s at 94 °C, 90 s at 60 °C, 90 s at 72 °C, and then a final extension of 30 min at 60 °C. The PCR products were diluted with 200 µL dH_2_0. For all samples, 0.5 µL of diluted product was loaded in 15 µL HIDI formamide with 0.1 µL LIZ-500 size standard (ThermoFisher Scientific, Waltham, MA, USA) and electrophoresed using an ABI 3130XL Genetic Analyzer (ThermoFisher Scientific, Waltham, MA, USA). We scored and binned genotypes using Genemapper v5.0 (ThermoFisher Scientific, Waltham, MA, USA).

### Genetic diversity analyses

#### Microsatellite Loci

We used GenAlEx v6.5 to determine the number of alleles, observed heterozygosity (H_o_), expected heterozygosity (H_E_), and F_IS_ at each locus in each taxon separately (Peakall & Smouse, 2006; Peakall & Smouse, 2012). We also used GenAlEx v6.5 to calculate F_ST_ and unbiased Nei’s genetic distance between species and subspecies. Because the magnitude of F_ST_ is influenced by heterozygosity, we also present the standardized measure F′_ST_ developed by Meirmans & Hedrick (2011). Microsatellite loci were tested for deviations from Hardy–Weinberg Equilibrium (HWE) with heterozygote excess, as well as genotypic linkage equilibrium using GENEPOP v4.7 (Rousset, 2008). We used a sequential Bonferroni correction to account for multiple comparisons in these tests. Null alleles were identified using MICROCHECKER v2.2.3 (Van Oosterhout et al., 2004), and then loci with null alleles and significant deviations from HWE were removed from further analyses. HP-RARE was used to calculate allelic richness (A_r_) using rarefaction to consider the differences in sample sizes between taxa (Kalinowski, 2005). When amplifying microsatellite loci cross-species, it is possible to lose variability across loci which would lead to an underestimate of nuclear genetic diversity. One expectation of ascertainment bias at microsatellite loci is that the median allele size is expected to be smaller in the species for which the loci were not originally developed since shorter microsatellites are generally less variable (Crawford et al., 1998). We compared the median allele sizes for the loci used in yellow bat species to median allele sizes of the same loci in *L. borealis* and *A. cinereus* using Mann–Whitney U tests. For *D. ega* and *D. intermedius* there was no difference in median allele lengths with those of *L. borealis* and *A. cinereus*, suggesting ascertainment bias is not strong in these species (*D. ega* versus *L. borealis*/*A. cinereus* medians: 269 bp and 266 bp, *W* = 84.0, *P* = 0.93; *D. intermedius* versus *L. borealis*/*A. cinereus* medians: 274 bp and 267 bp, *W* = 40, *P* = 0.94).

#### Mitochondrial DNA

We calculated haplotype diversity (*h*) in GenAlEx v6.5 and nucleotide diversity of mitochondrial haplotypes (*π*) using DnaSP v6 (Rozas et al., 2017). Mitochondrial COI sequences from putative *D. intermedius* subspecies sampled in this study and downloaded from GenBank were used to create a Minimum Spanning Network in PopArt (Leigh & Bryant, 2015).

### Population structure

We tested for evidence of population structure for each taxon individually, for *D. i. floridanus* and *D. i. intermedius* together, and for *D. ega* with *D. intermedius* combined using STRUCTURE v2.3.4, which clusters multilocus microsatellite genotypes based on the number of genetically distinct populations (Pritchard, Stephens & Donnelly, 2000). We assumed admixture, correlated allele frequencies, and omitted prior taxon designation. We used the Markov Chain Monte Carlo for 10^6^ iterations after a burn-in period of 10^4^ iterations for 10 replicates of *K* = 1–5 clusters. STRUCTURE can give misleading results for the number of populations and individual ancestry if there is uneven sampling across clusters, K (Puechmaille, 2016; Wang, 2017). To mitigate these potential biases, we used the recommendations of Wang (2017) and set the prior for admixture to allow *α* to vary between clusters and we decreased the initial *α* from 1.0 to 0.2. We estimated the most likely K using the method from Evanno, Regnaut & Goudet (2005) and by determining the highest LnP(D) before values plateaued (Pritchard, Stephens & Donnelly, 2000). We used CLUMPP v1.1.1 to average the most likely K across ten replicate runs (Jakobsson & Rosenberg, 2007). We considered individuals to be admixed between clusters when their ancestry (q) was ≥ 0.10 in each of two or more clusters, a value that has been used in a number of other studies (Vähä & Primmer, 2006; Barilani et al., 2007; Sanz et al., 2009; Bohling, Adams & Waits, 2013; Johnson et al., 2015).

### Population expansion and effective population size

We tested for neutrality using DnaSP v6 in each taxon and used the COI sequences to calculate Fu’s F and Tajima’s D (Fu, 1997; Tajima, 1989). Values showing significant negative deviations from the null model of a stable population indicate historical population growth. Historic female effective population size (N_Ef_) was estimated by first calculating Watterson’s estimator of COI sequence diversity (*θ*) in Arlequin v3.5, and then by using the equation: *θ*= 2N_e_*u*, where *u* is the mutation rate per sequence per generation (Excoffier & Lischer, 2010; Schenekar & Weiss, 2011). As the mutation rate of the COI gene is not known for either yellow bat species, we used mutation rates of the cytochrome b gene from other bat species of Vespertilionidae (Nabholz, Glémin & Galtier, 2008). The high and low mutation rates used were 9.115 × 10^−5^ and 6.751 × 10^−6^ per sequence per year, respectively. Contemporary effective population size (N_e_) was estimated from the microsatellite genotypes using NeEstimator v2.1, and a minimum allele frequency of 0.05 was used to calculate upper and lower limits of N_e_ with the linkage disequilibrium model assuming random mating (Do et al., 2014).

## Results

### *Dasypterus ega* - microsatellite genetic diversity

After genotyping 119 *D. ega* individuals at 13 microsatellite loci, we removed 4 loci due to either null alleles or deviations from HWE. None of the remaining nine loci exhibited a heterozygote deficit or genotypic linkage disequilibrium. One sample was removed after failing to amplify at >50% of the loci. One hundred fifteen individuals amplified successfully at all 9 loci (Table 1). Observed heterozygosity (H_o_) ranged from 0.513 to 0.974 across loci (mean 0.760 ± 0.050 SE), with the number of alleles ranging from 2 to 40. Allelic richness (A_r_) ranged from 3.14 to 4.61 (3.548 ± 0.329).

**Table 1:** Characterization of microsatellite loci for each *Dasypterus* taxon examined. Each locus has the number of individuals (n), allele size range in bp, number of alleles (N_*a*_), allelic richness (A_r_), observed heterozygosity (H_O_), expected heterozygosity (H_E_), and the inbreeding coefficient F_IS_. A_r_ was only calculated using loci shared among all taxa.

**Species**	**Locus**	**n**	**Size range (bp)**	**N**_a_	**A**_r_	**H**_O_	**H**_E_	**F**_IS_
*D. ega*	Coto_G12	118	211–231	10	–	0.839	0.808	−0.038
	LAS7468	115	311–419	40	4.61	0.974	0.954	−0.021
	LAS8830	117	254–284	13	3.59	0.821	0.822	0.002
	LAS9555	116	441–489	16	–	0.793	0.836	0.051
	Cora_F11	117	146–155	2	–	0.513	0.498	−0.029
	LAS9151	118	262–306	11	2.64	0.551	0.588	0.063
	LAS7831	116	401–423	11	3.76	0.871	0.849	−0.026
	LcM	116	195–241	20	–	0.690	0.712	0.031
	LcU	116	220–253	7	3.14	0.793	0.750	−0.057
*D. i. floridanus*	Coto_G12	50	211–231	10	–	0.800	0.839	0.047
	LAS7468	49	325–376	20	4.41	0.980	0.927	−0.057
	LAS8830	50	262–276	8	3.19	0.760	0.751	−0.012
	LAS8425	50	166–172	4	–	0.300	0.269	−0.117
	LAS9151	50	272–290	4	2.00	0.520	0.491	−0.059
	LAS7831	50	399–418	10	3.82	0.800	0.854	0.063
	LcM	50	203–245	13	–	0.740	0.803	0.078
	LcU	50	228–253	7	2.99	0.640	0.662	0.033
*D. i. intermedius*	LAS7468	208	325–384	24	4.37	0.938	0.930	0.932
	LAS8830	211	264–278	8	3.23	0.716	0.764	−0.009
	LAS2547	184	428–461	15	–	0.842	0.827	0.063
	LAS8425	211	166–188	5	–	0.209	0.202	−0.019
	LAS9151	211	264–293	9	2.10	0.507	0.519	−0.032
	LAS7831	205	397–417	11	3.94	0.873	0.876	0.022
	LcU	211	224–228	7	2.99	0.720	0.715	0.004
*D. intermedius*	LAS7468	257	325–384	37	4.52	0.946	0.946	0.001
	LAS8830	261	262–278	9	3.22	0.724	0.762	0.050
	LAS8425	261	166–188	5	–	0.226	0.215	−0.051
	LAS9151	261	264–293	9	2.08	0.510	0.513	0.008
	LAS7831	255	397–418	12	3.93	0.859	0.876	0.019
	LcU	261	224–253	9	2.96	0.705	0.708	0.004

### *Dasypterus intermedius* - microsatellite genetic diversity

A total of 267 individuals identified by DNA barcoding as either of the two *D. intermedius* subspecies (50 *D. i. floridanus* and 217 *D. i. intermedius*) were genotyped at 13 microsatellite loci. For *D. i. floridanus*, we removed 5 loci due to null alleles or deviations from HWE. Forty-nine individuals amplified at all loci (Table 1). Observed heterozygosity (H_o_) ranged from 0.300 to 0.980 across loci (0.692 ± 0.073), with the number of alleles ranging from 4 to 20. Allelic richness (A_r_) ranged from 2 to 4.41 (3.282 ± 0.406). For *D. i. intermedius*, we removed 6 loci due to null alleles or deviations from HWE. One hundred eighty-four individuals amplified at all loci, and we removed 5 samples after failing to amplify at > 50% of loci (Table 1). Observed heterozygosity (H_o_) ranged from 0.209 to 0.938 (0.686 ± 0.096), with the number of alleles ranging from 5 to 24. Allelic richness (A_r_) ranged from 2.1 to 4.37 (3.362 ± 0.393). And finally, we also analyzed both *D. intermedius* subspecies together at 6 shared loci (255 individuals amplified at all loci; Table 1). Observed heterozygosity (H_o_) ranged from 0.226 to 0.946 (0.662 ± 0.106), with the number of alleles ranging from 5 to 37. Allelic richness (A_r_) ranged from 2.08 to 4.52 (3.342 ± 0.418).

### Mitochondrial genetic diversity

**Figure 1:**
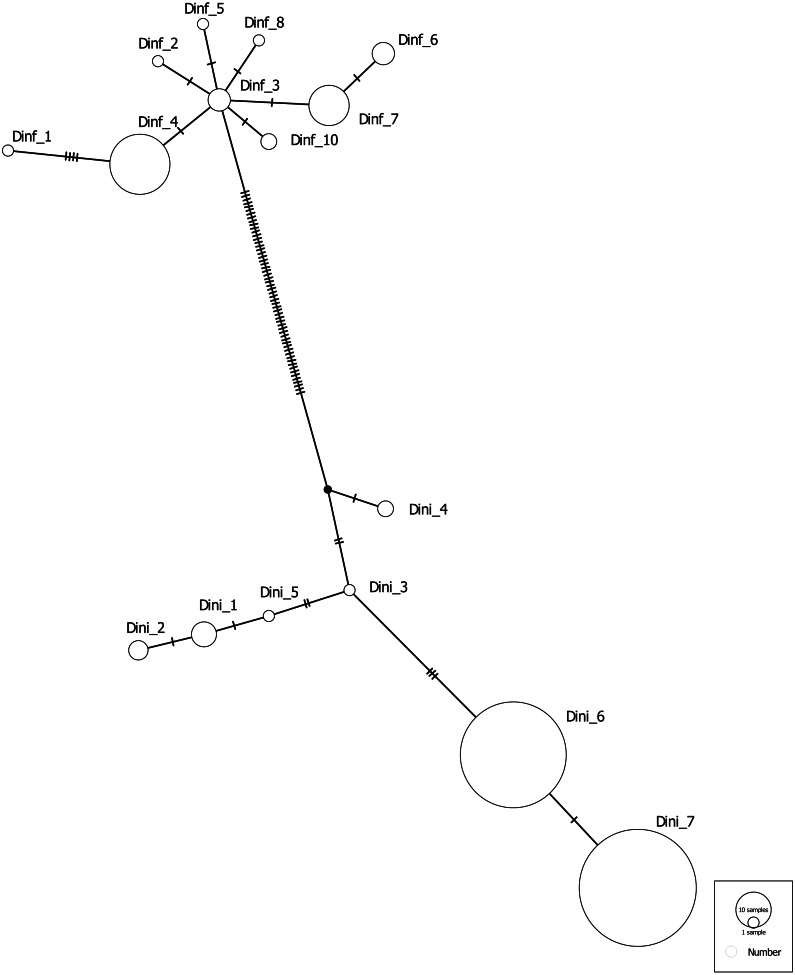
Minimum spanning haplotype network of unique mitochondrial COI sequences. Minimum spanning haplotype network of unique COI sequences from *Dasypterus i. floridanus* (Dinf) and *D. i. intermedius* (Dini) individuals from this study and from GenBank. Circles indicate haplotypes and the size of each circle corresponds to the number of individuals having that haplotype. Vertical hatch marks represent the number of nucleotide substitutions between haplotypes.

We identified two unique haplotypes in 112 *D. ega* individuals (Table 2). The most common haplotype had a frequency of 0.991 and nucleotide diversity (*π*) was 0.00003. We found six unique haplotypes in 50 *D. i. floridanus* individuals, with the most common haplotype having a frequency (*h*) of 0.580. Seven unique haplotypes were found in 212 *D. i. intermedius* (Table 2). The most common haplotype had a frequency of 0.527. In *D. i. floridanus* nucleotide diversity was 0.00225, whereas in *D. i. intermedius* it was 0.00177. The minimum spanning haplotype network for *D. intermedius* revealed two clusters separated by 56 base substitutions which corresponded to *D. i. floridanus* and *D. i. intermedius* (Fig. 1).

**Table 2:** Characterization of mitochondrial diversity for each *Dasypterus* taxon examined. Number of individuals (n) of each taxon analyzed for mtDNA diversity using number of unique haplotypes (H), haplotype diversity (h), and nucleotide diversity (*π*). mtDNA sequences were also used to calculate Tajimas D, Fu’s Fs, and Watterson’s estimator (theta).

**Taxon**	**n**	**H**	**h**	*π*	**Tajima’s D**	**Fu’s F**_s_	*θ*
*D. ega*	112	2	0.018	0.00003	−1.011	−2.344	0.188
*D. i. floridanus*	50	6	0.588	0.00225	−0.821	−0.369	1.786
*D. i. intermedius*	203	7	0.542	0.00177	−0.825	−0.663	1.522

### Population structure

**Figure 2:**
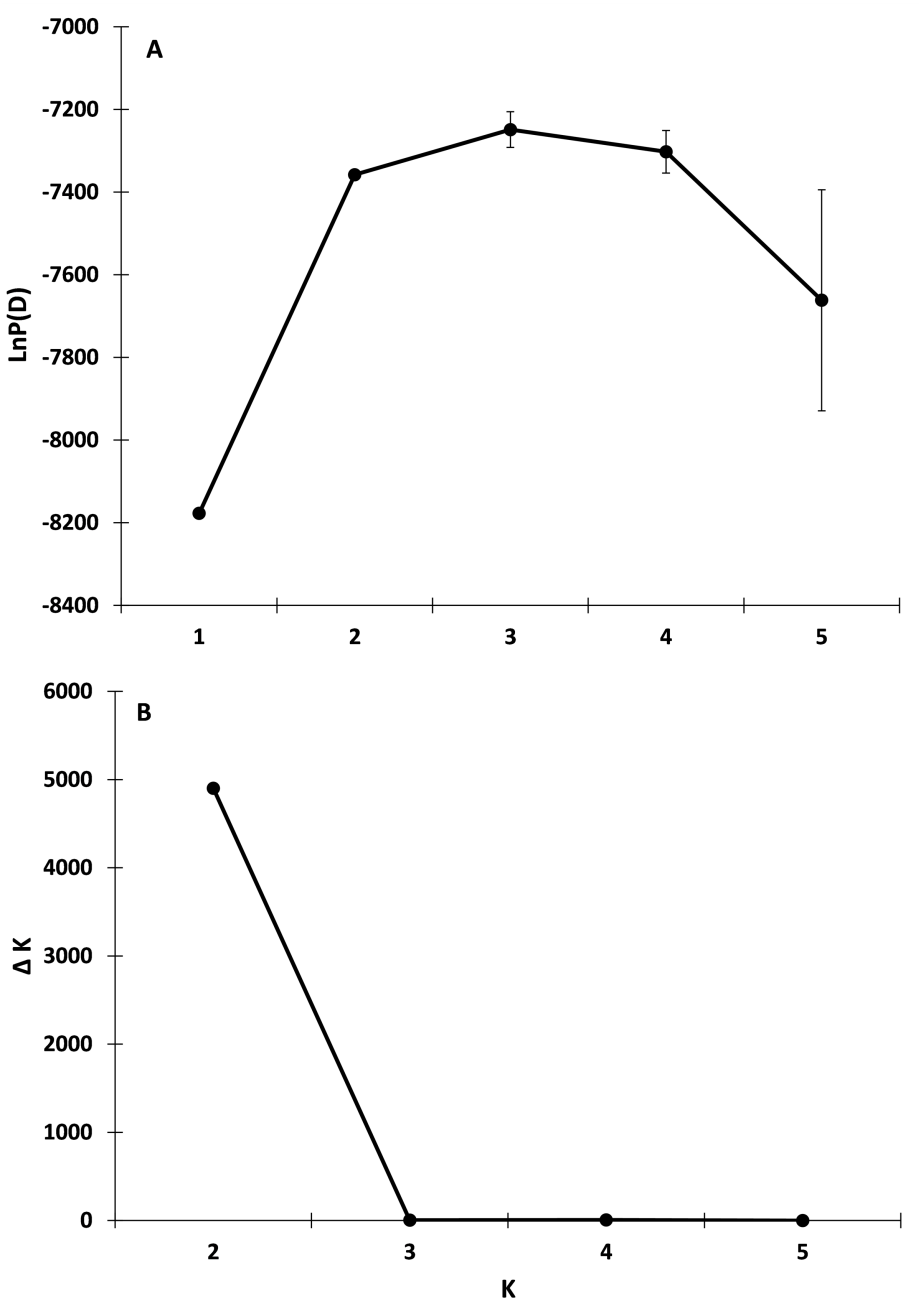
Most likely number of populations as calculated by STRUCTURE for all bats. (A) Mean of Ln estimated probability of data ±  SD. (B) Delta K from Evanno, Regnaut & Goudet (2005).

**Figure 3:**
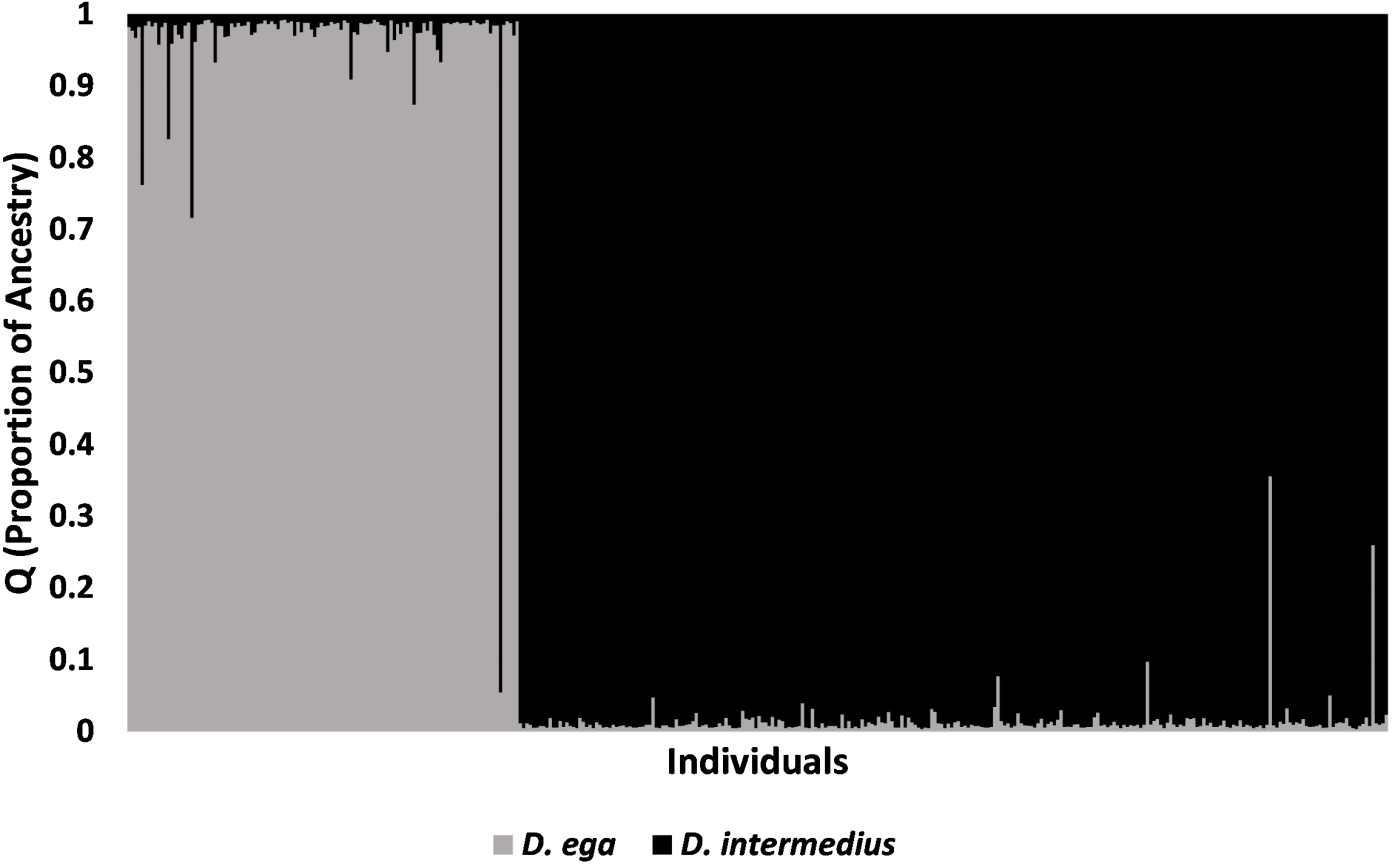
STRUCTURE plot showing the proportion of ancestry for all *Dasypterus* bats. STRUCTURE plot for *K* = 2 showing the proportion of ancestry for *D. ega* and *D. intermedius*. Vertical bars with both colors indicate individuals of mixed ancestry.

Genetic differentiation between *D. ega* and *D. intermedius* was high once values were corrected for heterozygosity (F_ST_ = 0.13, *P* = 0.001; F′_ST_ = 0.610, Nei’s = 0.788). There was low but significant genetic differentiation at microsatellite loci that increased slightly after correcting for heterozygosity between *D. i. floridanus* and *D. i. intermedius* (*F*_ST_ = 0.014, *P* = 0.001; F′_ST_ = 0.057, Nei’s = 0.046). The STRUCTURE analyses indicated that the most likely number of genetic clusters within each of the three taxa was *K* = 1. When *D. i. floridanus* and *D. i. intermedius* were analyzed together, the most likely number of clusters (K) was one. When *D. ega* and *D. intermedius* were analyzed together, the LnP(D) method provided evidence of *K* = 3 as the number of clusters with the highest support; however, it was apparent that the largest change of LnP(D) was between *K* = 1 and 2, which more often corresponds to the true K (Pritchard, Wen & Falush, 2010; Fig. 2A). The Evanno, Regnaut & Goudet (2005) method also identified *K* = 2 as the best-supported number of clusters when *D. ega* and *D. intermedius* were analyzed together. At *K* = 2, *D. ega* and *D. intermedius* belonged to separate genetic clusters (Figs. 2B and 3). The STRUCTURE analysis provided some evidence of hybridization between *D. ega* and *D. i. intermedius* (Fig. 3). One individual with a *D. ega* mtDNA haplotype had 95% ancestry with *D. intermedius*, whereas four other individuals with *D. ega* haplotypes had on average 21% ancestry (13–24%) with *D. intermedius.* There were two individuals with *D. intermedius* mtDNA haplotypes that had 64% and 74% ancestry, respectively, with *D. ega* (Fig. 3).

### Population expansion and effective population size

Tajima’*s* D and Fu’s F were −1.011 and −2.344 in *D. ega*, −0.821 and -0.369 in *D. i. floridanus* and −0.825 and −0.663 in *D. i. intermedius*, respectively (Table 2). None of the values were significantly different from zero and therefore do not support a history of past demographic expansion. Estimates of N_Ef_ were lower for *D. ega* than *D. intermedius*, whereas estimates for the two *D. intermedius* subspecies were similar (Table 3). Contemporary estimates of N_e_ all had upper bounds of infinity. Similar to estimates of N_Ef_, the point estimates of N_e_ and the lower 95% CI of N_e_ was lower for *D. ega* than *D. intermedius*.

## Discussion

### Genetic diversity and effective population size

Both mitochondrial and nuclear microsatellite genetic diversity were lower in *D. ega* and *D. intermedius* compared to two closely related species, *A. cinereus* (H_o_ = 0.86, *h* = 0.78–0.87) and *L. borealis* (H_o_ = 0.81–0.82, *h* = 0.95–0.99; Vonhof & Russell, 2015; Korstian, Hale & Williams, 2015; Pylant et al., 2016). There are three non-mutually exclusive hypotheses why genetic diversity may be lower in yellow bats. First, both *A. cinereus* and *L. borealis* mate while undertaking seasonal migration, leading to gene flow between geographically distanced populations which would maintain high nuclear and matrilineal genetic diversity (Cryan et al., 2012; Vonhof & Russell, 2015; Korstian, Hale & Williams, 2015; Pylant et al., 2016). In contrast, yellow bats are thought to be non-migratory and so local populations like the ones we sampled may not have as high of diversity as these other species (Baker, Mollhagen & Lopez, 1971; Decker et al., 2020). Second, the lower genetic diversity found in our study could also be due to genetic drift since our study site is located at a geographic range-edge for both taxa (Webster, Jones & Baker, 1980; Decker et al., 2020). The samples collected at our study site may therefore only represent a subset of the genetic diversity found at the range-center of these species (Eckert, Samis & Lougheed, 2008; Cahill & Levinton, 2015). Third, the lower diversity we observed in yellow bats compared to *A. cinereus* and *L. borealis* may be due to ascertainment bias as the microsatellite loci used in this study were developed for use in those other species (Li & Kimmel, 2013). Median allele sizes in *D. ega* and *D. intermedius* were similar to what was observed in *A. cinereus* and *L. borealis* for the loci used in this study; suggesting that ascertainment bias at microsatellite loci is not strong for these species.

**Table 3:** Estimated effective population size for each *Dasypterus* taxon examined. Estimated current effective population size (N_e_) with 95% confidence intervals and estimated historic female effective population size (N_Ef_) using low and high mutation rates for each taxon in this study.

**Taxon**	**n**	**N**_**e**_	**Lower 95% CI**	**Upper 95% CI**	**N**_**Ef**_**Low**	**N**_**Ef**_**High**
*D. ega*	118	423	169	∞	1,031	13,924
*D. i. floridanus*	50	∞	551	∞	9,797	132,277
*D. i. intermedius*	212	1,032	325	∞	8,349	112,724
*D. intermedius*	262	2,880	396	∞	–	–

Estimates of historic (long-term) effective population size for yellow bats were similar to *A. cinereus* (10^3^–10^4^) but lower than *L. borealis* (10^5^–10^6^; (Vonhof & Russell, 2015; Korstian, Hale & Williams, 2015; Pylant et al., 2016). Estimates of current Ne have confidence limits that broadly overlap those from *A. cinereus* and *L. borealis* from those previous studies. Estimating contemporary N_e_ is difficult for species with N_e_ > 1,000 using the LD method (Waples & Do, 2010). The upper limits to the 95% confidence intervals were infinite for all taxa. To achieve greater confidence in estimates of N_e_, future studies will require many more additional genetic markers (Waples & Do, 2010). Nonetheless, the lower bound to the 95% confidence interval may still be informative as to the occurrence of bottlenecks if N_e_ is repeatedly estimated as part of a genetic monitoring protocol for yellow bats over time (Waples & Do, 2010; Korstian, Hale & Williams, 2015). We recommend future population-genetic studies of bats use next generation sequencing methods with many more markers (e.g., SNPs) to increase the power of conclusions inferred from genetic data.

### Population structure and expansion

Both *D. ega* and *D. intermedius* have geographic ranges that extend along the Gulf Coast and south beyond the border with Mexico into Central America (and into South America for *D. ega*; Barquez & Diaz, 2016; Miller & Rodriguez, 2016). *D. ega* also appears to be expanding northward following plantings of ornamental palm trees, their preferred roost, and so they may be a more recent colonist of south Texas (Spencer, Choucair & Chapman, 1988; Demere et al., 2012; Decker et al., 2020). Using STRUCTURE, we found no evidence of population substructure in any of the three yellow bat taxa which might be expected since we may be sampling non-migratory populations at the edge of their ranges. *D. i. floridanus* has a star-shaped minimum-spanning haplotype network, which is expected under a scenario of past population range expansion (Avise, 2000); however, Tajima’s D and Fu’s F were not significantly different from zero for any of the taxa, providing no statistical evidence of past demographic population expansion.

### Taxonomy

Both the mitochondrial locus and microsatellite loci clearly distinguish *D. ega* and *D. intermedius* and both F′_ST_ and Nei’s genetic distance are high between the two species. We found evidence that hybridization sometimes occurs between *D. ega* and *D. intermedius* in south Texas and this deserves further study to determine if hybridization occurs throughout the region of sympatry between these two species in Mexico and Central America or if it is occurring primarily at their range edges. Bats are thought to have lower rates of cross-species hybridization than some other taxa, although an increasing number of studies are reporting evidence for hybridization, which has probably played an important role in speciation (e.g., Bogdanowicz, Piksa & Tereba, 2012; Çoraman et al., 2019).

Using mtDNA, we found two monophyletic clades that corresponded to the two subspecies of *D. intermedius* (Chipps et al., 2020; Decker & Ammerman, 2020; Fig. 1); and yet, when we compared microsatellite genotypes between these two clades we found little differentiation. These results are consistent with two other recent studies that also found little to no genetic differentiation at either a nuclear intron or microsatellite loci (Decker, 2019; Decker & Ammerman, 2020). Differentiation as measured by *F*_ST_ (∼0.01) between these groups was significant but also below the lower threshold at which STRUCTURE can distinguish between groups (F_ST_∼0.03–0.05 or an F′_ST_ of ∼0.30; Latch et al., 2006). The five loci that were used in these analyses were fairly polymorphic (average H_E_ = 0.75; average number of alleles = 10–11) and easily discriminated between *D. intermedius* and *D. ega* and so it seems unlikely that low diversity *per se* may have resulted in low differentiation between these groups.

There are several possibilities for the low divergence at nuclear loci, including hybridization following secondary contact and incomplete lineage sorting. A previous study of these subspecies has suggested the mito-nuclear discordance is most likely due to secondary contact and hybridization (Decker & Ammerman, 2020), which would be consistent with our results and evidence for morphological intergradation between the subspecies. Populations isolated for long periods of time can become differentiated at both mitochondrial and nuclear loci due to adaptation and genetic drift, although it is expected that mtDNA will complete the process of lineage sorting before nuclear DNA since it has an effective size $ \frac{1}{4} $ that of the nuclear genome (Hudson & Turelli, 2003; Zink & Barrowclough, 2008; Toews & Brelsford, 2012). Future studies will need to sample *D. intermedius* across its entire range and use a larger suite of markers to determine the most likely cause of the mito-nuclear discordance we see in this region of overlap in Texas.

### Conservation genetics and wind energy development

Mitigating the impacts of climate change by increasing electricity generation from renewable sources like wind power has the potential to benefit wildlife conservation (e.g., Kiesecker et al., 2011; Allison et al., 2019). Nonetheless, there are increasing concerns that wind energy development could cause declines in bat populations (e.g., Kunz et al., 2007; Erickson et al., 2016; Frick et al., 2017). For most bat species, however, we lack estimates of population sizes which makes it exceedingly difficult to place this new source of mortality in context. Thus, researchers are increasingly turning to intrinsic markers like stable isotopes and genetic characteristics to estimate population parameters, identify migratory pathways, and measure demographic trends (e.g., Lehnert et al., 2014; Korstian, Hale & Williams, 2015; Pylant et al., 2016), with the intent of reducing uncertainty regarding the impacts of wind energy on bat populations. With respect to genetic data, for example, we know that when formerly large populations are reduced to small sizes, genetic diversity is lost, the possibility of inbreeding depression increases, and deleterious variants can accumulate through fixation such that individual and population fitness are reduced (Frankham, Ballou & Briscoe, 2009; Koepfli & Gooley, 2020). Therefore, understanding the amount of genetic diversity within wild populations and how it is distributed among geographic populations is a major concern for conserving the integrity and sustainability of species.

To ascertain whether certain bat species are at risk of suffering from negative effects associated with reduced population size and low genetic diversity, several recent studies have used population genetic approaches to elucidate patterns of genetic variation, identify potential barriers to gene flow, and estimate N_e_ for three migratory tree bats killed at wind energy facilities across North America. For *L. borealis*, studies estimating N_e_ have concluded that the effective population size is in the hundreds of thousands to millions of individuals, with no evidence of barriers to gene flow among groups of samples, suggesting *L. borealis* is one panmictic population (Korstian, Hale & Williams, 2015; Vonhof & Russell, 2015; Pylant et al., 2016; Sovic, Carstens & Gibbs, 2016). Similarly for *A. cinereus*, there is no evidence of population genetic structure (Korstian, Hale & Williams, 2015; Pylant et al., 2016; Sovic, Carstens & Gibbs, 2016); although the estimates of N_e_ are smaller for this species (ranging from several thousand to hundreds of thousands of individuals) compared to *L. borealis* (Pylant et al., 2016). For *L. noctivigans*, Sovic, Carstens & Gibbs (2016) found no evidence for population structure in this species, although it had the smallest estimated N_e_ of the three species. Collectively, based on the results from these studies, we would predict that *L. borealis* would be somewhat more resilient to population declines caused by wind turbine mortality compared to *A. cinereus* and *L. noctivigans*. Furthermore, the high levels of gene flow and connectivity across the population ranges of these migratory tree bats indicate that monitoring and management efforts must integrate information from across their entire ranges as the potential impacts of mortality in any given region may have far-reaching implications. To date, there is no strong genetic evidence of population declines in these species; however, data from other sources suggest their populations may be decreasing (e.g., Winhold, Kurta & Foster, 2008; Ford et al., 2011; Francl et al., 2012; Frick et al., 2017; Rodhouse et al., 2019).

The results from this study indicate that genetic diversity is lower in both *Dasypterus* species compared to what has been reported for the other tree bat species studied to date using similar methods (e.g., Korstian, Hale & Williams, 2015; Pylant et al., 2016). If these populations are in fact non-migratory, this would limit gene flow with populations in other parts of their range and reduce to the likelihood of demographic rescue via immigration if wind turbine mortality were to cause local population declines. Currently the migratory habits of *D. ega* and *D. intermedius* are poorly known and thus warrant further investigation. *D. intermedius* is thought to be non-migratory, roosting year-round in Spanish moss or in palm trees in Texas (Decker et al., 2020). Similarly, *D. ega* is thought to be a year-round resident in far-south Texas, roosting in ornamental palm trees (Schmidly & Bradley, 2016), but it may be migratory in other parts of its range (Ammerman, Hice & Schmidly, 2012). Thus, due to uncertainty around the migratory status and population sizes of *Dasypterus* species, additional data should be collected to determine if there is the potential for wind energy development to affect persistence of local *Dasypterus* populations. We therefore recommend that periodic population genetic assessment take place in order to gauge the impacts of wind turbine mortality on these species.

## Conclusions

Our study provides population genetic data for two species of yellow bats in the Lower Rio Grande River Valley of Texas, and demonstrates the utility of continuing to use lower cost methods such as microsatellites and mtDNA sequence data to investigate genetic variation in new species of interest for which genomics tools are not yet available (Koepfli & Gooley, 2020). We do recommend, however, that future efforts should focus on developing genomic resources, obtaining better estimates of mutation rates, and conducting range-wide population genetic studies to better estimate historical and current population sizes of tree bat species impacted by wind energy development. By increasing the number of markers, genomics will provide greater power to estimate and monitor N_e_, identify population bottlenecks, improve identification of evolutionarily significant units and management units, and may provide additional power to detect more subtle patterns of differentiation and estimate demographic patterns and parameters with greater precision (Allendorf, Hohenlohe & Luikart, 2010). We also recommend that genetic monitoring be continued over time as a means of assessing the impacts of wind turbine mortality on bat populations (Schwartz, Luikart & Waples, 2007). With baseline genetic data that will allow us to assess population status and trends, we will improve our chances of developing sound conservation and mitigation strategies for this important and diverse vertebrate group.

##  Supplemental Information

10.7717/peerj.10348/supp-1 Supplemental Information 1 Sequence alignment of unique COI sequences Alignment used for minimum spanning haplotype network of unique COI sequences from *Dasypterus i. floridanus* (Dinf) and *D. i. intermedius* (Dini) individuals from this study and from GenBank.

10.7717/peerj.10348/supp-2 Supplemental Information 2 Microsatellite genotypes and mitochondrial COI haplotypes for *Dasypterus ega* and *D. intermedius*

## Additional Information and Declarations

### Field Study Permissions

The following information was supplied relating to field study approvals (i.e., approving body and any reference numbers): Texas Parks and Wildlife Department (TPWD SPR-0213-023) Duke Energy and EDP Renewables (EDPR 0320007188)

### DNA Deposition

The following information was supplied regarding the deposition of DNA sequences: The mitochondrial cytochrome oxidase I sequences are available at GenBank: MT510982, MT510983, and MT510986 to MT510998.

### Data Availability

The following information was supplied regarding data availability: Raw data is available in the Supplemental Files.
